# In Vitro Evaluation of Oxidative Stress Induced by Oxime Reactivators of Acetylcholinesterase in HepG2 Cells

**DOI:** 10.1021/acs.chemrestox.3c00203

**Published:** 2023-11-11

**Authors:** Nela Váňová, L’ubica Múčková, Tereza Kalíšková, Lukáš Lochman, Petr Bzonek, František Švec

**Affiliations:** † Department of Pharmaceutical Chemistry and Pharmaceutical Analysis, Faculty of Pharmacy in Hradec Králové, Charles University, Akademika Heyrovského 1203, Hradec Králové 500 05, Czechia; ‡ Department of Toxicology and Military Pharmacy, Faculty of Military Health Sciences, University of Defence, Třebešská 1575, Hradec Králové 500 02, Czechia; § Department of Analytical Chemistry, Faculty of Pharmacy in Hradec Králové, Charles University, Akademika Heyrovského 1203, Hradec Králové 500 05, Czechia

## Abstract

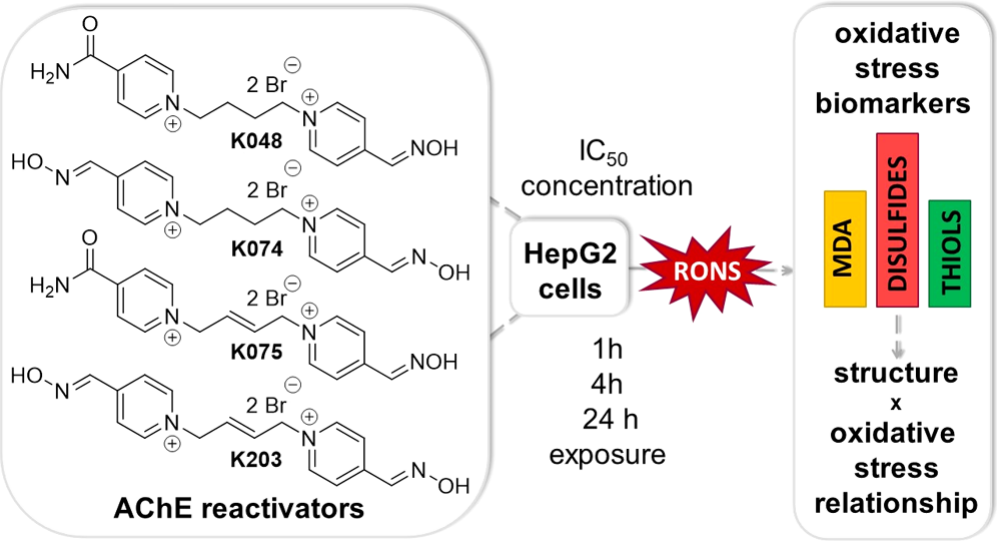

Oxime reactivators of acetylcholinesterase (AChE) are used as causal antidotes for intended and unintended poisoning by organophosphate nerve agents and pesticides. Despite all efforts to develop new AChE reactivators, none of these drug candidates replaced conventional clinically used oximes. In addition to the therapeutic efficacy, determining the safety profile is crucial in preclinical drug evaluation. The exact mechanism of oxime toxicity and the structure–toxicity relationship are subjects of ongoing research, with oxidative stress proposed as a possible mechanism. In the present study, we investigated four promising bispyridinium oxime AChE reactivators, K048, K074, K075, and K203, and their ability to induce oxidative stress *in vitro*. Cultured human hepatoma cells were exposed to oximes at concentrations corresponding to their IC_50_ values determined by the MTT assay after 24 h. Their potency to generate reactive oxygen species, interfere with the thiol antioxidant system, and induce lipid peroxidation was evaluated at 1, 4, and 24 h of exposure. Reactivators without a double bond in the four-carbon linker, K048 and K074, showed a greater potential to induce oxidative stress compared with K075 and K203, which contain a double bond. Unlike oximes with a three-carbon-long linker, the number of aldoxime groups attached to the pyridinium moieties does not determine the oxidative stress induction for K048, K074, K075, and K203 oximes. In conclusion, our results emphasize that the structure of oximes plays a critical role in inducing oxidative stress, and this relationship does not correlate with their cytotoxicity expressed as the IC_50_ value. However, it is important to note that oxidative stress cannot be disregarded as a potential contributor to the side effects associated with oximes.

## 1 Introduction

The high acute toxicity and lethality of organophosphate (OP) nerve agents or pesticides increase the importance of developing an effective antidotal therapy to address a broad spectrum of OP poisoning. From a pharmacological perspective, conventional treatment of OP poisoning includes the live-saving intravenous application of atropine followed by the administration of the reactivator of OP-inhibited acetylcholinesterase (AChE) and symptomatic treatment with diazepam. Despite intensive efforts devoted to the structure design and synthesis of new oxime-based AChE reactivators, none of these compounds replaced or supplemented the clinically most relevant oximes, pralidoxime and obidoxime, over the past 60 years.^1^ The importance of preclinical testing of newly prepared drugs lies in evaluating their therapeutic efficiency and assessing their safety profile.

Numerous drug candidates from a group of reactivators called ″K-oximes″ have been intensively studied regarding their *in vitro* and *in vivo* activity. However, they still did not fulfill the desired criteria in terms of a broad reactivation profile and potency. Nevertheless, some of these compounds, e.g., K027, K048, and K203 oxime, exhibited promising reactivation potential against individual OP preserving acceptable toxicity at therapeutical doses.^2−5^ Although the relationship between the biological activity of oximes, i.e., the reactivation efficacy, and their chemical structure was well-studied,^6−8^ elucidation of the mechanism of toxicity requires further research. One possible mechanism of oxime toxicity discussed is the oxidative damage to key biomolecules in living organisms.^9^

**Figure 1:**
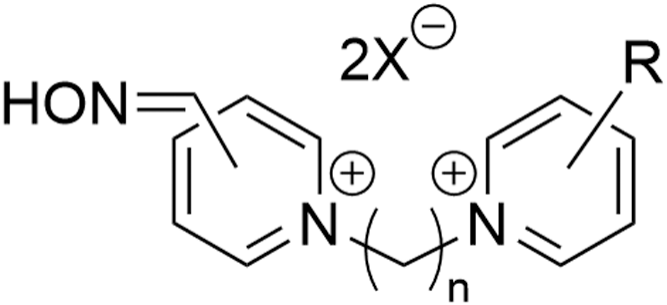
General structure of bisquarternary oxime reactivators of AChE.

In a recent *in vitro* study by Muckova et al., the ability of structurally diverse oxime reactivators, including pralidoxime (2-PAM), methoxime (MMB-4), asoxime (HI-6), obidoxime (LüH-6), and trimedoxime (TMB-4), to induce oxidative stress was examined. The findings revealed that quaternary oxime reactivators with the functional aldoxime group at position 4 of the pyridinium ring were more potent inducers of oxidative stress than compounds with the aldoxime group at position 2. Interestingly, the length of the connecting chain or the incorporation of oxygen in it had an insignificant or minor impact on oxidative stress induction (Figure 1). Based on the current knowledge of the structural features influencing the activity and toxicity of AChE reactivators, as well as their ability to affect redox homeostasis, four bispyridinium oximes, K048, K074, K075, and K203 with promising activity, were selected to investigate this phenomenon further. The relationship between the cytotoxicity of oximes, expressed as the IC_50_ value, and the severity of oxidative stress has not been fully elucidated.^10,11^ However, the thorough characterization of specific structural features responsible for inducing oxidative stress holds notable value in the design and development of highly effective OP-antidotal drugs,^9^ especially when oxidative stress has emerged as a potential contributor to serious drug-induced side effects, which in the past led even to the postmarket withdrawal of drugs.^12^ The primary objectives of this study were to assess the impact of the number of aldoxime groups (one or two) attached to the bispyridinium skeleton of the AChE reactivator, evaluate the influence of substituting one aldoxime group with a carbamoyl group, and investigate the effect of the presence or absence of a but-2(*E*)-en-1,4-diyl linker between the pyridinium moieties on the induction of oxidative stress in the HepG2 cell line. This will be achieved by measuring the levels of reactive oxygen and nitrogen species (RONS) with fluorescent probes and by chromatographic determination of malondialdehyde (MDA), nonprotein thiols (NP-SH), and nonprotein disulfides (NP-SS-NP).

## 2 Materials and Methods

### 2.1 Chemicals for Oxidative Stress Induction

All tested oximes, namely, K048 [4-carbamoyl-1-(4-(4-((hydroxyimino)methyl)pyridin-1-ium-1-yl)butyl)pyridin-1-ium dibromide], K074 [1,1′-(butane-1,4-diyl)bis(4((hydroxyimino)methyl) pyridine-1-ium) dibromide], K075 [1,1′-((*E*)-but-2-ene-1,4-diyl)bis(4-((hydroxyimino)methyl) pyridin-1-ium)dibromide], and K203 [4-carbamoyl-1-((2*E*)-4-(4-((hydroxyimino)methyl)pyridin-1-ium-1-yl)but-2-en-1-yl)pyridin-1-ium dibromide] were provided by the Department of Toxicology and Military Pharmacy (Faculty of Military Health Sciences, University of Defense, Hradec Kralove, Czech Republic). For their structures, see Figure 6. The purity of tested oximes was assessed by high-performance liquid chromatography-ultra-violet (HPLC-UV) under the chromatographic conditions described by Vanova et al.,^13^ reaching 97.8, 99.1, 96.1, and 99.1% for K048, K074, K075, and K203, respectively. *Tert*-butyl peroxide (TBHP; Luperox DI) was purchased from Sigma-Aldrich (St. Louis, MO).

### 2.2 Cell Line

The human hepatoma cell line (HepG2) is a well established cell line used for cytotoxicity screening of oximes enabling high-throughput analysis. This cell line is characterized by its low levels of phase I and II enzymes, which result in decreased metabolic activity. Consequently, this attribute allows the evaluation of the toxic effects exerted by the parent compound.^10,14,15^ The HepG2 cell line (HB-8065, ATCC, Manassas, VA) was cultivated in high-glucose Dulbecco’s modified Eagle’s medium (DMEM; Biosera, Nuaille, France) supplemented with 10 vol % fetal bovine serum (Biosera, Nuaille, France) and a 0.1 vol % penicillin–streptomycin antibiotic solution (Sigma-Aldrich, St. Louis, MO) at 37 °C and 5% CO_2_ in a humidified incubator (Binder CO_2_ Incubator CB 160, Tuttlingen, Germany). After the HepG2 cells reached about 80% confluence, they were harvested using a 0.025% trypsin/EDTA solution (Sigma-Aldrich, St. Louis, MO), and the cell suspension was transferred into a new 75 or 25 cm^2^ culture flask or seeded into 96-well plates (TPP Techno Plastic Products, AG, Trasadingen, Switzerland).

### 2.3 Colorimetric Cell Viability Assay

The toxicological indices IC_50_ used in the present study were measured utilizing 3-(4,5-dimethylthiazol-2-yl)-2,5-diphenyl-tetrazolium bromide (MTT) reduction assay after 24 h of incubation with tested compounds. For the assay, HepG2 cells were seeded into 96-well plates in a 100 μL volume and density of 15 × 10^3^ cells per well. Cells were allowed to attach overnight before the treatment. The stock solutions of tested compounds were prepared and serially diluted in DMEM. The concentration ranges of tested compounds were as follows: 2–250 μmol/L for TBHP, 12.5 μmol/L–200 mmol/L for K048 and K074 oximes, and 0.625 μmol/L–50 mmol/L for K075 and K203 oximes. After 24 h of incubation, the cultivation medium containing serially diluted substances was aspirated and replaced with a fresh medium containing MTT at a concentration of 0.5 mg/mL and subsequently incubated at 37 °C for 1 h. The medium with MTT was then aspirated, and formazan was dissolved in 100 μL of dimethyl sulfoxide (Sigma-Aldrich, St. Louis, MO). The optical density of all wells was measured using a Spark multimode microplate reader (Tecan Group Ltd., Männedorf, Switzerland) at 570 nm. Each experiment was carried out in triplicate and repeated three independent times. The concentrations of oximes and TBHP (positive control) corresponding to their IC_50_ values will be used to detect RONS by fluorescent probes and induce oxidative damage in HepG2 cells.

### 2.4 Detection of Reactive Oxygen and Nitrogen Free Radicals

Two different fluorescent dyes, i.e., 2,7-dichlorodihydrofluorescein diacetate (DCFH-DA, Cayman Chemicals Company, Ann Arbor, MI) and dihydroethidium (DHE, Sigma-Aldrich, St. Louis, MO) were utilized for the determination of intracellular levels of RONS after 1, 4, and 24 h of incubation with tested oxime reactivators or TBHP (positive control). Cells incubated with oxime-free DMEM represented untreated control. After the incubation, the experimental medium was removed and replaced with a solution of DCFH-DA or DHE at concentrations of 20 and 5 μmol/L, respectively. The cells were subsequently incubated at 37 °C in a CO_2_ incubator for 45 min. Afterward, the fluorescence intensity of each well was measured using a Spark multimode microplate reader at an excitation wavelength of 485 nm for DCFH-DA or 528 nm for the DHE probe and an emission wavelength of 535 or 590 nm, respectively. Each experiment was carried out in triplicate and repeated three independent times.

### 2.5 In Vitro Induction of Oxidative Stress

The culture medium was removed from a 25 cm^2^ culture flask on the day of the experiment, and cells were washed twice with 3 mL of phosphate-buffered saline (PBS; GE Healthcare Life Sciences, South Logan, UT). Subsequently, 3 mL of a plain DMEM medium (negative control) and a DMEM medium containing TBHP (positive control) or individual oximes were added to each flask, and cells were incubated for 1, 4, and 24 h. The concentration of individual oxime and TBHP in the DMEM medium corresponded to the IC_50_ value. After incubation, the experimental medium was removed, and cells were washed with 3 mL of PBS and harvested by scraping. The resulting suspension was centrifuged at 220*g* and 21 °C for 5 min (Universal 320R centrifuge, Hettich, Tuttlingen, Germany). The supernatant was removed and dried cell pellets were stored at −80 °C. Each experiment was carried out in three independent replicates.

### 2.6 Preparation of the HepG2 Cell Homogenate for the Chromatographic Determination of Malondialdehyde, Nonprotein Thiols, and Nonprotein Disulfides

The cell pellets were thawed at room temperature, resuspended in 500 μL of ultrapure water (Millipore Purification System, Merck, Millipore, Darmstadt, Germany), and sonicated for 2 min (2 s cycle, amplitude 20%) in a Q500 homogenizer (QSONICA Sonicators, Newton). Then, the homogenate was divided into three parts: 250 μL for the determination of malondialdehyde (MDA), 100 μL for each determination of nonprotein thiols (NP-SH) and disulfides (NP-SS-NP), and 10 μL for the Bradford protein assay.

### 2.7 LC-MS/MS Analysis of Total Intracellular MDA

#### 2.7.1 Sample Preparation

For the determination of total intracellular MDA levels, 250 μL of the HepG2 homogenate in a 1.5 mL microcentrifuge tube (Eppendorf, Hamburg, Germany) was spiked with 10 μL of 10 μmol/L d^2^-MDA (internal standard) synthesized from 1,1,3,3-tetraethoxypropane-1,3-d2 (Cambridge Isotope Laboratories, Tewksbury), according to Tsikas.^16^ The sample was subjected to alkaline hydrolysis of protein-bound MDA at 60 °C in a heating block (DB-3 Sample Concentrator, TECHNE, Cole-Parmer, U.K.) for 30 min after the addition of 50 μL of 6 mol/L aqueous sodium hydroxide. After cooling on ice, the sample was acidified with 150 μL of 35% trichloroacetic acid (v/v) and centrifuged at 3500*g* at 4 °C for 10 min (IEC CL31R Multispeed Centrifuge, Thermo Fisher Scientific, San Jose). Then, 400 μL of the supernatant was transferred into a new 1.5 mL microcentrifuge tube and mixed with 25 μL of a 25 mmol/L 2,4-dinitrophenylhydrazine (DNPH) solution prepared in 2% formic acid (FA) in acetonitrile (ACN; VWR, Leuven, Belgium) v/v. The mixture was incubated at 37 °C and 300 RPM for 60 min in a Thermomixer Comfort (Eppendorf) protected from light. The derivatized mixture was cleaned via solid-phase extraction (SPE; Visiprep 24DL manifold, SUPELCO, Bellefonte) using Phenomenex STRATA C18-E 100 mg/1 mL (55 μm, 70 Å) cartridges (Phenomenex, Torrance). The SPE cartridge was conditioned with 1 mL of methanol (MeOH; JT Baker, Avantor, Gliwice, Poland), equilibrated with 1 mL of water, and after the sample was loaded, it was washed with 700 μL of water and the analytes were eluted with 1 mL of MeOH. The eluent was evaporated under the stream of nitrogen at 60 °C and reconstituted in 100 μL of 70% MeOH (v/v), and 20 μL of the sample was injected into the liquid chromatography–mass spectrometry/MS (LC-MS/MS) system. If not specified differently, all chemicals and reagents were purchased from Sigma-Aldrich/Merck (Darmstadt, Germany).

#### 2.7.2 Quantification of MDA

Calibrators for six-point calibration curve construction were prepared by a spiking blank homogenate of HepG2 cells with an MDA stock solution (50 μmol/L) at a concentration range from 0.1 to 2.0 μmol/L (including zero calibrator) and with 10 μL of the internal standard. Samples were then processed as described in Section 2.7.1.

#### 2.7.3 LC-MS/MS Analysis

Samples were analyzed using a Shimadzu Prominence HPLC system consisting of the DGU-20A Prominence Degasser, the LC-220AT Liquid Chromatography Pump, the CTO-20A Column Oven, and the SIL-20A Prominence Autosampler (Shimadzu, Kyoto, Japan) coupled with a Thermo Finnigan LCQ Advantage Max Ion Trap mass spectrometer equipped with the atmospheric pressure chemical ionization (APCI) probe (Thermo Fisher Scientific). The chromatographic separation was achieved using a Phenomenex KINETEX C18(2) column (150 mm × 3 mm, 2.6 μm, 100 Å) protected with a Security Cartridge (4 mm × 2 mm; Phenomenex, Torrance). The analysis was carried out at 40 °C and a constant flow rate of 0.270 mL/min with the mobile phase consisting of 0.1% aqueous FA (v/v, A) and MeOH (B) under the following gradient shape: 0–1 min 50–75% B, 1–7 min 75% B, 7.1 min 50% B, and 7.1–11 min 50% B. The APCI source operated in a positive mode and was set as follows: source heater temperature: 325 °C, sheath gas: 65 arb, aux gas: 20 arb, discharge current: 5 μA, capillary temperature: 275 °C, capillary voltage: 14 V, and tube lens offset: −10 V. Data were acquired in the selected reaction monitoring mode (SRM) with ion transitions *m*/*z* [M + H]^+^ 235 → 159, 189 for MDA and *m*/*z* [M + H]^+^ 237 → 161, 191 for d^2^-MDA. The method was validated according to the European Medicines Agency (EMA) Guideline on Biomedical method validation. For the full description of the validation procedure and results, see Supporting Information, page S2, Table S-1.

### 2.8 HPLC-UV Analysis of Intracellular NP-SH and NP-SS-NP

The HepG2 cell homogenate obtained, as described in Section 2.6, was processed for the determination of intracellular NP-SH and NP-SS-NP, according to Muckova et al.^11^ The samples were analyzed using the HPLC system described above equipped with an SPD 20-AV Prominence UV/vis detector. The analysis was carried out on a Phenomenex LUNA C18 column (150 mm × 3 mm, 3 μm, 100 Å) protected with a Security Cartridge (4 mm × 2 mm, Phenomenex, Torrance) at 30 °C and a constant flow rate of 0.330 mL/using the mobile phase consisting of 0.9% aqueous FA (A) and ACN (B) under gradient conditions: 0–1 min 12% B, 1.1–2 min 12–55% B, 2–7 min 55% B, 7.1 min 55% B, and 7.1–10 min 12% B. The detector was set at 326 nm and the sample injection volume was 20 μL.

### 2.9 Statistical Analysis

The toxicological indices IC_50_ were calculated from control-subtracted triplicates using nonlinear regression (four parameters) using GraphPad Prism 9 software version 9.3.0 (GraphPad Software Inc., San Diego, CA). One-way analysis of variance (ANOVA) followed by Dunnetʼs multiple comparison test was used for RONS, MDA, NP-SH, and NP-SS-NP statistical analysis by GraphPad Prism 9 software, version 9.3.0. Data are expressed as means ± standard deviation (SD) of three independent measurements (*n* = 3). Significant differences between oxime-treated and untreated control groups (*p* ≤ 0.05) were marked by an asterisk (*).

## 3 Results

### 3.1 Determination of Toxicological Index IC_50_

The cytotoxicity of AChE reactivators was evaluated using a colorimetric MTT assay. The cells were exposed to the tested compounds for 24 h. The cytotoxicity of each reactivator expressed as IC_50_ is shown in Table 1. The less toxic compound was K048, with an IC_50_ of 30.60 mmol/L. The cytotoxicity of oxime reactivators of AChE increased from K074 toward K075 and K203, with IC_50_ being 1.13-, 12.6-, and 14,9-fold lower, respectively. The TBHP, used as a positive control for oxidative stress induction, was the most toxic. The percentage of intact, early, and late apoptotic and necrotic HepG2 cells after 24 h incubation with oximes was determined using flow cytometric analysis to confirm the IC_50_ values from the MTT assay. The description of the procedure and results are provided in the Supporting Information, pages S2–S4, Figure S-1.

**Table 1:** Half Minimal Inhibitory Concentration (IC_50_) of Tested Compounds

compound	IC_50_ [mmol/L]
K048	30.60
K074	27.18
K075	2.43
K203	2.05
TBHP	0.13

### 3.2 Detection of RONS

**Figure 2:**
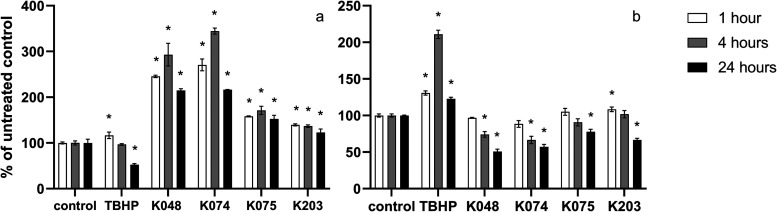
Changes in intracellular levels of RONS in HepG2 cells determined using DCFH-DA (a) and DHE (b) fluorescent probe after 1 h (white column), 4 h (gray column), and 24 h (black column) treatment with oxime AChE reactivators (*n* = 3). Results are expressed in % of RONS of untreated controls (cells incubated with the oxime-free DMEM). One-way analysis of variance (ANOVA) followed by Dunnetʼs multiple comparison test was used for statistical analysis. Significant differences between oxime-treated and untreated control groups are indicated: * (*p* ≤ 0.05).

The level of intracellular RONS was detected using the fluorescent probe DCFH-DA (Figure 2a) and DHE (Figure 2b) at three different time intervals (1, 4, and 24 h) and compared to negative controls. After a 1 h incubation with the tested compounds, the amount of RONS detected using the DCFH-DA probe significantly increased as follows: by 16.7% in TBHP, 146% in K048, 171% in K074, 58.0% in K075, and 39.2% in K203-treated cells. When the DHE was utilized, an increase of 30.7% in free radicals in TBHP-treated cells and an 8.5% increase in K203-treated cells were observed compared to the negative control. After 4 h, RONS levels measured with DCFH-DA were higher as follows: by 193% in K048, 244% in K074, 71.2% in K075, and 39.2% in K203-treated cells. Using the DHE dye, the intracellular levels of RONS were increased after 4 h in cells exposed to TBHP by 111%, K048 by 25.8%, and K074 by 33.4%. After 24 h, the RONS levels were decreased by 47.6% in cells exposed to TBHP but increased by 115% in K048, 117% in K074, 52.4% in K075, and 22.8% in K203-treated cells, when measured by the DCFH-DA probe, and by 22.8% in TBHP, 49.2% in K048, 42.8% in K074, 33.5% in K075, and 22.1% in K203 exposed cells when determined by DHE probe.

### 3.3 Intracellular Levels of MDA

**Figure 3:**
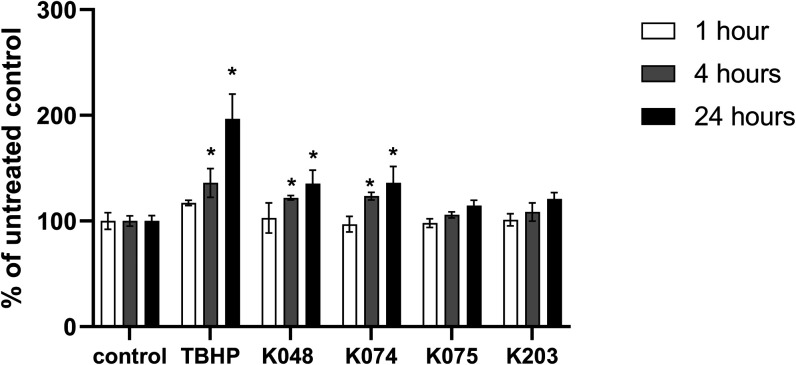
Changes in intracellular levels of MDA in HepG2 cells determined by LC-MS/MS after 1 h (white column), 4 h (light gray column), and 24 h (dark gray column) of treatment with oxime AChE reactivators (*n* =). Results are expressed in % of RONS of untreated controls (cells incubated with the oxime-free DMEM). One-way analysis of variance (ANOVA) followed by Dunnetʼs multiple comparison test was used for statistical analysis. Significant differences between oxime-treated and untreated control groups are indicated: * (*p* ≤ 0.05).

MDA is one of the most commonly reported and reliable oxidative stress biomarkers resulting from the peroxidation of polyunsaturated fatty acids mainly localized in cell membranes. Excessive oxidative damage to membrane lipids strongly disturbs its integrity and leads to cell death.^17^ Significant changes in MDA levels (Figure 3) were found only for K048 and K074 oxime after 4 and 24 h treatments. MDA concentration was also significantly elevated in cells exposed to TBHP (4 and 24 h). After 4 h incubation, MDA levels were higher by 36.1% in TBHP, 17.2% in K048, and 23.6% in K074-treated cells. After 24 h of exposure, the tested compounds led to a 96.6% increase in MDA levels in TBHP, 35.4% in K048, and a 36.1% increase in K074-treated cells.

### 3.4 Intracellular Levels of NP-SH and NP-SS-NP

**Figure 4:**
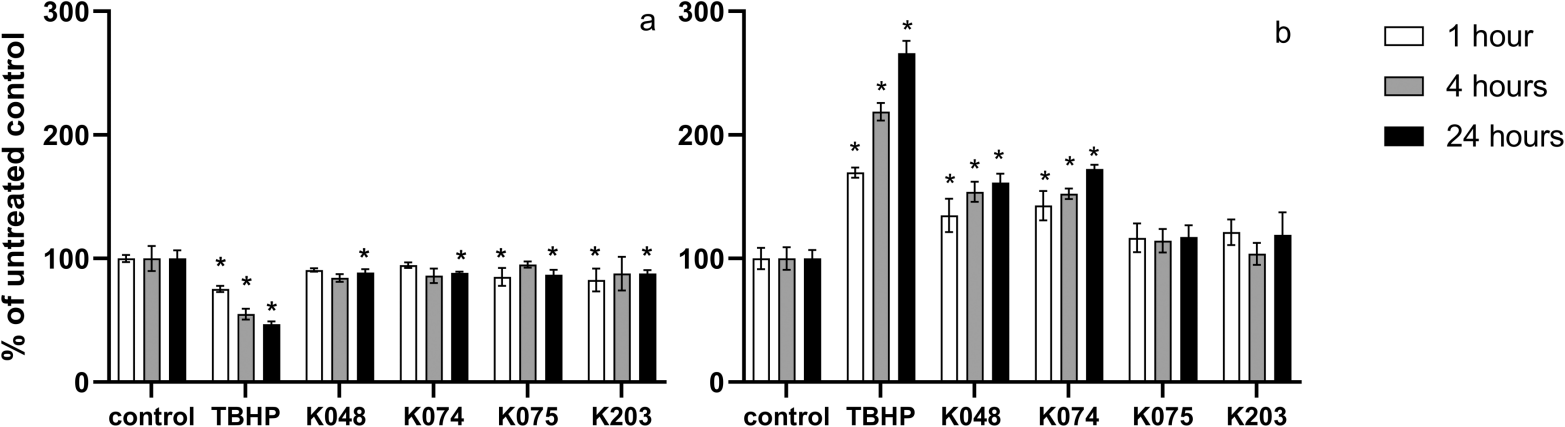
Changes in intracellular levels of NP-SH (a) and NP-SS-NP (b) in HepG2 cells determined by HPLC-UV after 1 h (white column), 4 h (light gray column), and 24 h (dark gray column) of treatment with oxime AChE reactivators (*n* = 3). Results are expressed in % of RONS of untreated controls (cells incubated with the oxime-free DMEM). One-way analysis of variance (ANOVA) followed by Dunnetʼs multiple comparison test was used for statistical analysis. Significant differences between oxime-treated and untreated control groups are indicated: * (*p* ≤ 0.05).

Alterations in thiol and disulfide levels impair intracellular antioxidant defense. The NP-SH component of the thiol redox state includes the most abundant thiol in mammalian cells, glutathione (GSH), and also homocysteine, cysteine, cysteine-containing low-molecular-weight peptides, or coenzyme A. The oxidized form of GSH, the glutathione disulfide, constitutes only a minor fraction of NP-SS-NP formed by oxidized forms of NP-SH and their mixed disulfides.^18^ In TBHP-treated cells, a significant decrease in NP-SH levels was observed, with reductions of 24.6, 45.0, and 53.1% after 1, 4, and 24 h, respectively. K048 and K074 oximes caused an 11.4% and an 11.6% decrease in NP-SH after 24 h of exposure. In cells treated with K075 and K203, NP-SH levels decreased by 14.8 and 17.4% after 1 h and by 13.1 and 12.1% after 24 h (Figure 4a). Additionally, TBHP treatment resulted in a substantial increase in NP-SS-NP levels, showing increases of 69.6, 119, and 166% after 1, 4, and 24 h. Exposure to K048 and K074 oximes also increased NP-SS-NP levels, with changes of 34.8 and 42.8% after 1 h, 54.8 and 52.4% after 4 h, and 61.5 and 72.3% after 24 h (Figure 4b).

## 4 Discussion

### 4.1 Chromatographic Determination of Oxidative Stress Biomarkers

In our present study, we improved and validated the LC-MS/MS method previously used for determining intracellular MDA levels.^19^ We accomplished this by replacing glutaraldehyde, a structural analogue of MDA used as an internal standard, with deuterated MDA. This replacement addressed several issues associated with the sample preparation step and chromatographic analysis. Using d_2_-MDA allowed us to increase the intensity of sample washing during the SPE procedure 3-fold without affecting MDA recovery. This, in turn, facilitated the more efficient removal of signal-suppressing components and interferents (e.g., remaining DNPH) and enabled the sample concentration before LC-MS/MS analysis. This approach significantly reduces the number of cells required for conducting *in vitro* structure/toxicity experiments.

### 4.2 Oxidative Stress Induced by Oxime Reactivators of AChE

Oximes are primarily evaluated for their therapeutic efficacy based on their ability to reactivate OP-inhibited AChE. The formation of various OP-AChE conjugates during both intended and unintended intoxication by diverse OP structures highlights the need for a broad-spectrum reactivator. Still, none of the newly developed compounds met this criterion. Despite several limitations, mono- and bisquarternary pyridinium aldoximes still represent leading structures in the design of new AChE reactivators and have been registered for clinical use. Several structural features have been suggested as being essential for the high reactivation ability of bispyridinium oximes. These include the presence of quaternary nitrogen, which mediates the reactivator’s affinity for inhibited AChE, at least one aldoxime group attached to the pyridinium ring (preferably at position 2 or 4), and rigidity in the connection chain along with a distance between pyridinium moieties (typically three to five equivalent C–C bond; Figure 1). However, some of these structural aspects that enhance the reactivator efficacy exhibit a degree of OP dependence, which complicates the search for a broad-spectrum reactivator. Furthermore, additional structural modifications aim to improve the penetration through the blood–brain barrier to reactivate brain AChE and counteract the lethal neurotoxic effects of OP.^20−22^ Besides the reactivation efficiency, structural modifications of AChE reactivators mentioned previously may also enhance their toxicity. In general, recent in vivo and *in vitro* investigations suggested that the number of aldoxime groups attached to the pyridinium moieties higher than two does not significantly improve the activity of the reactivator but can negatively affect its toxicity. Also, the oxime group in the para position on the pyridinium ring is preferred over the ortho position and is associated with reduced toxicity. A double bond in the connecting linker results in slightly higher toxicity compared to that of methylene linkages. Substituting one oxime group with a carbamoyl group positively influences reactivation with minimal effect on toxicity.^21−24^

**Figure 5:**
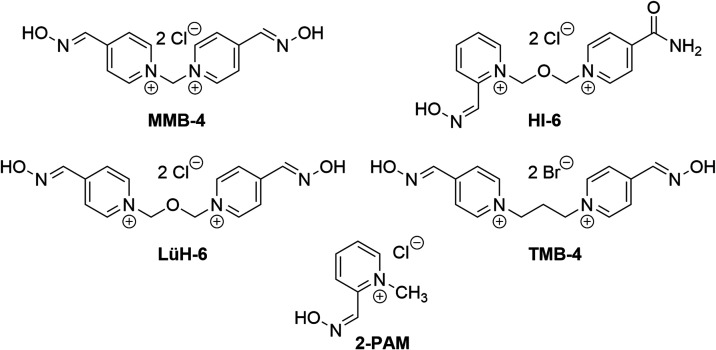
Chemical structure of conventional oxime reactivators of AChE: methoxime (MMB-4), asoxime (HI-6), obidoxime (LüH-6), trimedoxime (TMB-4), and pralidoxime (2-PAM).

**Figure 6:**
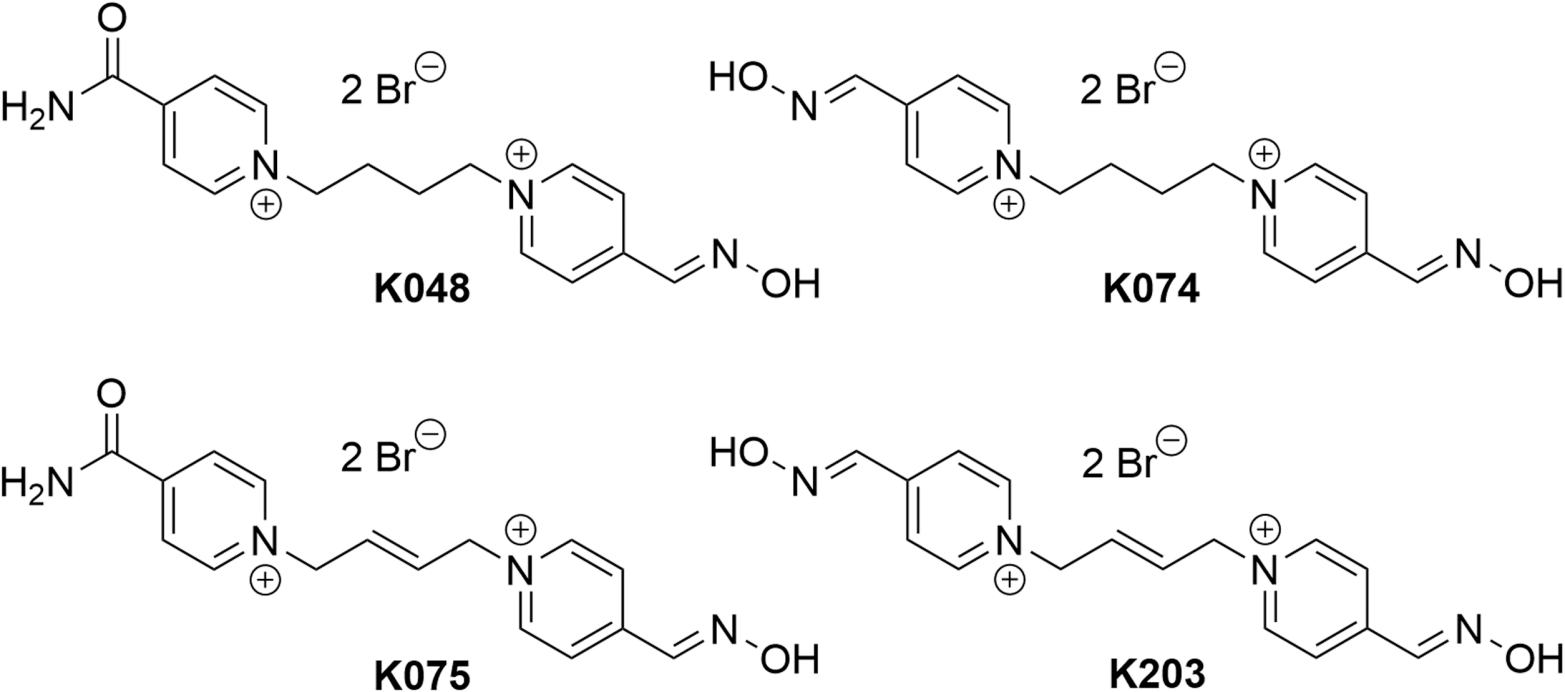
Chemical structures of K-oxime reactivators of AChE involved in the study.

All of the oximes selected in our study have been, to some degree, studied regarding their *in vitro* and *in vivo* toxic effects, aiming at explaining the mechanism of their toxicity. Conventional cell viability and cytotoxicity assays enable fast and cheap screening of newly synthetized compounds. During primary oxime cytotoxicity screening, the IC_50_ value is usually established on several cell lines, including HepG2 cells, SH-SY-5Y, or HK-2.^25,26^ The concentration of oximes used in this *in vitro* study (IC_50_ at 24 h) exerted varying effects on cell viability and induced observable cytotoxic effects in a time-dependent manner. This allowed us to monitor the dynamics of oxidative stress induction and its associated damage after 1 and 4 h of exposure. In HepG2 cells, the IC_50_ values for the tested oximes, ranging from 2 to 30 mM, did not exceed the values obtained for conventional oximes, which ranged from 2 to 20 mM.^11^ However, it is essential to note that concentration, or the IC_50_ value, is not the only factor determining toxicity. Thus, understanding the mechanisms behind the toxicity of these compounds is of great importance. In a previous study involving K048, K074, and K075 oximes, a significant positive correlation was observed between their IC_50_ values (representing the concentration required to inhibit AChE activity by 50%) and their LD_50_ values. This IC_50_ value also correlated with the capacity to mitigate the relative risk of mortality in the presence of the organophosphate paraoxon when measured in rat blood. The higher IC_50_ value of K048 oxime in rat blood (∼643 μmol/L) indicates its lower intrinsic AChE inhibitory activity and aligns with the higher IC_50_ value observed in HepG2 cells (∼30 mmol/L). Conversely, K074 (IC_50_ ∼ 66 μmol/L in rat blood) and K075 (IC_50_ ∼ 101 μmol/L in rat blood) exhibited similar activities, but IC_50_ values determined in HepG2 cells were approximately 27 and 2.4 mmol/L, respectively.^27^ In the study by Zandona et al., the toxicity of various bispyridinium oximes was investigated to understand their *in vitro* mechanism of toxicity. Oximes containing a but-2(*E*)-en-1,4-diyl linker and chlorine substitution exhibited higher toxicity across different cell lines and concentration ranges. When SH-SY5Y cells were exposed to K867 and K870 oximes (mono- and bis-chlorinated analogues of K203 oxime) for 4 h, there was no LDH leakage, indicating the preservation of cell membrane integrity. These findings suggest that these structures may induce regulated cell death, which was further confirmed by detecting specific apoptosis markers and the activation of caspase-9, initiating the intrinsic mitochondrial-dependent apoptotic pathway.^28^ In caspase-9-dependent apoptosis, RONS serve as essential mediators in initiating caspase activation alongside the release of mitochondrial cytochrome *c*. RONS directly oxidatively modify caspase-9 and apoptotic peptidase activating factor 1 (Apaf-1), thereby activating caspase-9, −3, and −10. The permanent charge and hydrophilic nature of bispyridinium oximes (indicated by a negative log *P* value) make it highly unlikely for oximes to penetrate the cell membrane (K048 log *P* −2.61, K074 log *P* −1.74, K075 log *P* −2.02, K203 log *P* N/A). This implies that apoptosis is the consequence of the oxime interaction with outer cell components, such as inhibition of the growth factor receptor (GFR), as postulated by Zandona et al. These pathophysiological events can also be modulated by oxidative stress.^28−31^ As shown in the Supporting Information (pages S2-S4 and Figure S-1), apoptotic cell death was also prevalent in HepG2 cells exposed to an IC_50_ concentration of K048, K074, K075, and K203 oximes for 24 h. The potential of oxime reactivators to modulate oxidative stress and explore its role in subsequent *in vitro* and *in vivo* processes has been the subject of several studies.^9 ,11 ,32 −36^ Despite these attempts to establish a connection between oxidative stress and the toxic effects of oximes, both *in vitro* and *in vivo*, oxidative stress does not seem to be the primary contributor to their toxicity. However, a link can be found between the structural aspects of oximes and the intensity of oxidative stress. The number and position of functional aldoxime groups and the nature of connecting linkers are the leading structural features responsible for AChE reactivation efficiency and thus have become the main subject of the structure–oxidative stress induction relationship investigation. In general, the numbers and position of aldoxime groups on the pyridinium moiety appear to affect their ability to initiate oxidative damage rather than the nature of the connecting chain. The most potent oxidative stress inductors, LüH-6, TMB-4, and MMB-4, bear two para-positioned aldoxime groups but differ by the length and nature of the connecting chain: methylene (MMB-4), propylene (TMB-4), and oxapropylene (LüH-6). The other two mono-oximes, monopyridinium 2-PAM and bispyridinium HI-6, with an *ortho*-position oxime moiety exhibited a lower impact on intracellular redox homeostasis (Figure 5). Despite the fact that the insertion of oxygen into the carbon linker in HI-6 (IC_50_ ∼ 3 mmol/L) and LüH-6 (IC_50 ∼_ 4 mmol/L) molecules does not seem to affect their ability to induce oxidative stress, both compounds exerted similarly high cytotoxicity in HepG2 cells. Additionally, the least toxic compound from this group, TMB-4, with a propylene linker (IC_50_ ∼ 22 mmol/L), showed comparable and, in some cases, slightly higher potency in inducing oxidative stress compared to the most toxic reactivator, MMB-4, with a methylene linker (IC_50_ ∼ 1 mmol/L).^10,11^ However, a critical structural factor that needs assessment regarding oxidative stress is the presence of a double bond in the connecting linker. This feature is absent in conventional reactivators but present in promising antidote candidates, such as K075 and K203. To assess the impact of the double bond on *in vitro* oxidative stress induction, four bispyridinium oximes, K048, K074, K075, and K203 (structures shown in Figure 6), were selected, each combining the presence of one or two aldoxime groups, the substitution of one aldoxime group with a carbamoyl group, and the presence of a double bond in the four-carbon connecting chain. We evaluated not only the *in vitro* generation of RONS by individual oximes using two fluorescent probes, DHE and DCFH-DA, but also their capacity to maintain thiol redox homeostasis and prevent resulting lipid peroxidation damage, which is crucial for cell membrane integrity and the regulation of cell death.

The HepG2 cell line was utilized to investigate the relationship between the structure of the oxime reactivators of acetylcholinesterase and oxidative stress. HepG2 cells are commonly used experimental models for screening the cytotoxicity of novel oximes and assessing drug-induced oxidative stress.^37,38^ The cells were exposed to the IC_50_ concentration of the oximes for 1, 4, and 24 h, and their response to oxidative stress was evaluated. The stability of the selected oximes in DMEM at 37 °C over a 24 h period was assessed to confirm sufficient oxime concentration throughout the entire experiment (Supporting Information, page S4, Figure S-2). The short-term duration of 4 h was chosen based on the anticipated peak generation of RONS within a 24 h time frame, during which approximately half of the exposed cells undergo apoptosis.

Different levels of RONS were detected in HepG2 cells exposed to TBHP and K-oximes while determined by DCFH-DA and DHE probes. DCFH-DA is a nonspecific fluorescent probe for determining various RONS, including hydrogen peroxide, hydroxyl radicals, organic peroxyl radicals, and peroxynitrite. It does not monitor the superoxide radical (O_2_^•–^), which is assessed using the DHE probe. The presence of a double bond in the connecting chain significantly enhanced the cytotoxicity of K075 and K203 (IC_50_ ∼ 2 mmol/L). Despite the lower IC_50_ value for these two oximes, their cytotoxicity does not correlate with their low potential to induce RONS production or to cause oxidative damage. While there was a slight increase in RONS generation by K075 and K203 observed using both DCFH-DA and DHE probes (accompanied by a modest reduction in NP-SH levels), the resulting MDA and NP-SS-NP levels within the 24 h time frame appear to be insignificant. Conversely, K048 (IC_50_ ∼ 30 mmol/L) and K074 (IC_50_ ∼ 27 mmol/L) oximes generated a significant increase in RONS production (except for O_2_^•–^), resulting in depletion of NP-SH and elevation of MDA and NP-SS-NP levels. Based on these results, both cytotoxicity and the potential to induce oxidative stress of selected oximes are defined by the presence or absence of the double bond in the connecting linker rather than by the number of functional aldoxime groups. Interestingly, the presence of a double bond affects cytotoxicity and oxidative stress induction in opposite ways.

Oximes are primarily designed as reactivators of OP-inhibited AChE, and organophosphates are known to induce significant oxidative stress. Therefore, if oximes possess no or low prooxidative properties, it would be beneficial for antidotal therapy if they could mitigate OP-induced oxidative stress. Selected oximes (K048, K074, K075, and K203) were previously tested for their potential antioxidant activity. In this experiment, HepG2 cells were simultaneously incubated for 120 min with TBHP (IC_50_ concentration) and oximes of the concentration described in the Supporting Information, page S5, Table S-2. These concentrations of oximes were approximately 10-fold lower than the IC_50_ values and did not affect the viability of HepG2 cells or increase the level of ROS production. The resulting ROS levels were measured using a DCF-DA fluorescent probe. We observed that from 15 min after simultaneous incubation with TBHP and K075 or K203, the ROS production was reduced by 80 and 98%, respectively. The ability of K075 and K203 to mitigate TBHP-induced oxidative stress *in vitro* was comparable to reduced glutathione (87%), the antioxidant used as a control. Slightly increased production of ROS was observed after combined exposure to TBHP with K048 (25%) and K074 (16%). This indicates the potential scavenging role of the double bond in K075 and K203 oximes toward ROS.^39^

K048 and K203 oximes were also studied in vivo to assess their impact on oxidative stress and its prevention. However, comparing and drawing conclusions from these studies are difficult due to variations in oxime doses, routes of administration, and observed oxidative stress biomarkers across different time intervals. For example, intraperitoneal administration of K048 at a dose of 25% LD_50_ (59.6 mg/kg) showed no signs of oxidative stress when evaluating thiobarbituric acid reactive substances (TBARS) and superoxide dismutase activity in rat plasma over a time range of 1 to 24 h. In the case of K203 oxime, an increase in low-molecular-weight antioxidants (LMWAs) was observed in plasma 180 min after intramuscular administration at a therapeutic dose (23 mg/kg) in rats, suggesting a potential benefit in nerve agent intoxication. However, the overall impact of postexposure administration of K203 oxime in protecting against oxidative stress induced by tabun was not significant.^35,40,41^

## 5 Conclusions

A study describing the relationship between the structure of selected oxime reactivators of AChE K048, K074, K075, and K203 and their potency to induce oxidative stress *in vitro* was conducted. While the results of the previous study with conventional AChE reactivators suggested that the induction of oxidative stress was influenced by the number and position of aldoxime groups in reactivators with a three-carbon-long connecting chain, our current study with reactivators having a four-carbon chain found that the presence of a double bond was the determining factor.

In conclusion, our findings underscore the structural factors that influence oximes in either inducing or mitigating oxidative stress, regardless of their cytotoxicity, as described by the IC_50_ value. The research aimed at elucidating the mechanism of toxicity of AChE reactivators is ongoing. While oxidative stress may not be the primary contributor, its potential involvement in oxime-related side effects cannot be disregarded and may be considered in alternative therapeutic approaches.

## Supporting Information Available

Validation of the LC-MS/MS method for determination of MDA in HepG2 cells, results of flow cytometric detection of cell death, stability of oximes in the DMEM medium at 37 °C over 24 h, and concentration of oximes used for the study of the antioxidant activity (PDF)

## Abbreviations

2-PAM: pralidoxime
AChE: acetylcholinesterase
ACN: acetonitrile
D^2^-MDA: deuterated malondialdehyde
DCFH-DA: 2,7-dichlorodihydrofluorescein diacetate
DHE: dihydroethidium
DMEM: Dulbecco’s modified Eagle’s medium
EMA: European Medicines Agency
FA: formic acid
GFR: growth factor receptor
GSH: glutathione
HepG2: human hepatoma cell line
HI-6: asoxime
K048: [4-carbamoyl-1-(4-(4-((hydroxyimino)methyl)pyridin-1-ium-1-yl)butyl)pyridin-1-ium dibromide]
K074: [1,1′-(butane-1,4-diyl)bis(4((hydroxyimino)methyl) pyridine-1-ium) dibromide]
K075: [1,1′-((*E*)-but-2-ene-1,4-diyl)bis(4-((hydroxyimino)methyl) pyridin-1-ium)dibromide]
K203: [4-carbamoyl-1-((2*E*)-4-(4-((hydroxyimino)methyl)pyridin-1-ium-1-yl)but-2-en-1-yl)pyridin-1-ium dibromide]
LMWA: low-molecular-weight antioxidants
LüH-6: obidoxime
MDA: malondialdehyde
MeOH: methanol
MMB-4: methoxime
MTT: 3-(4,5-dimethylthiazol-2-yl)-2,5-diphenyl-tetrazolium bromide
NP-SH: nonprotein thiols
NP-SS-NP: nonprotein disulfides
OP: organophosphates
PBS: phosphate-buffered saline
RONS: reactive oxygen and nitrogen species
SPE: solid-phase extraction
TBARS: thiobarbituric acid reactive substances
TBHP: *tert*-butyl hydroperoxide
TMB-4: trimedoxime
