# Supplementary material for: In Vitro Evaluation of Oxidative Stress Induced by Oxime Reactivators of Acetylcholinesterase in HepG2 Cells
Source: Chem Res Toxicol. 2023 Nov 11;36(12):1912–20. doi: 10.1021/acs.chemrestox.3c00203 (PMC10731658; doi:10.1021/acs.chemrestox.3c00203)
Supplement: Supplementary file 1 — tx3c00203_si_001.pdf [file tx3c00203_si_001.pdf]

Supporting Information

In vitro evaluation of oxidative stress induced by oxime reactivators of acetylcholinesterase in HepG2 cells

Nela Váňová[a*], Ľubica Múčková[b], Tereza Kalíšková[a], Lukáš Lochman[a], Petr Bzonek[b], František Švec[c]

[a]Department of Pharmaceutical Chemistry and Pharmaceutical Analysis, Faculty of Pharmacy in Hradec Králové, Charles University, Akademika Heyrovského 1203, Hradec Králové 500 05, Czechia

[b]Department of Toxicology and Military Pharmacy, Faculty of Military Health Sciences, University of Defense, Třebešská 1575, Hradec Králové 500 02, Czechia

[c]Department of Analytical Chemistry, Faculty of Pharmacy in Hradec Králové, Charles University, Akademika Heyrovského 1203, Hradec Králové 500 05, Czechia

*email: vanovan@faf.cuni.cz

Content of the Supporting Information:

1. Validation of LC-MS/MS method for determination of MDA in HepG2 cells
2. Flow cytometric detection of cell death
3. Stability of oximes in DMEM medium at 37 °C over 24 hours
4. Antioxidant activity of selected oxime reactivators of acetylcholinesterase

1. Validation of LC-MS/MS method for determination of MDA in HepG2 cells

The method was validated according to European Medicines Agency (EMA) Guideline on Biomedical method validation. Intra-day and inter-day accuracy and precision were determined by repeated measurements of HepG2 cell homogenate (section 2.6.) spiked with MDA at following concentrations: 0.1, 0.5, 1.0 and 1.5 μM MDA (n=5). The samples were processed as described in section 2.8. Recovery and matrix effect were evaluated by the post-extraction addition approach (n=2). Three sets of samples were prepared:

- Matrix-free standards prepared by derivatization of MDA solutions (in 70% MeOH) mixed with internal standard by 25 mM DNPH (ACN/FA, 98:2 v/v) to reach final MDA concentrations of 0.2 and 1.5 μM.
- "Pre-extracted" samples of blank cell homogenate spiked with MDA at concentrations 0.2 and 1.5 μM (n=2) before the procedure described in section 2.8.
- "Post-extracted" samples of blank cell homogenate processed as described in section 2.8. and subsequently spiked with pre-derivatized mixture of internal standard 0.2 and 1.5 μM MDA solution (n=2) after the SPE step.

Calculated parameters are shown in Table S-1.

Table S-1. Validation parameters of LC-MS/MS method for the determination of intracellular MDA

MDA concentration level (μmol/L)	0.1	0.2	0.5	1.0	1.5
Intra-day accuracy (%)	92.1 ± 5.0		95.4 ± 4.3	101.3 ± 4.2	94.7 ± 2.5
Inter-day accuracy (%)	108.6 ± 6.1		94.2 ± 2.7	102.4 ± 1.7	102.9 ± 2.6
Intra-day precision (%)	10.9		9.1	8.3	5.2
Intra-day precision (%)	11.3		5.7	1.6	5.0
Recovery (%)		85.6 ± 4.0			83.5 ± 4.5
IS-normalized matrix effect (%)		4.7			2.2

The accuracy and recovery are expressed as mean ± SD (n=5 or n=2). The precision (n=5) and internal-standard normalized matrix effect (n=2) are expressed as CV.

2. Flow cytometric detection of cell death

The microcapillary flow cytometry (Muse™ Cell Analyzer, Luminex, Austin, TX) was used to determine cell death. This method is based on detecting externalization of phosphatidylserine on the surface of cells undergoing apoptosis. The technique allows quantitative analysis of non-apoptotic, early apoptotic, late apoptotic, and necrotic cells. The reagent mixture also contains the membrane non-permeable dye 7-aminoactinomycin D. This component distinguishes necrotic and late apoptotic cells from the live and early apoptotic cells. The measurement of cell death was carried out according to manufacturer instructions after 1, 4, and 24 h incubation with tested oxime reactivators and with oxime-free medium as an untreated control. During the first day of the experiment, cells were seeded into a 24-well plate (TPP) with a density of 15 × 104. After the incubation, cells from each well were collected into the polystyrene tube (TPP) and centrifuged for 5 min at 1000 × g at room temperature. The supernatant was discarded. The cell suspensions

were prepared in fresh medium DMEM. Afterward 100 μL of cell suspension were mixed with the 100 μL of Muse™ Annexin V & Dead Cell Assay reagent, and the samples were incubated for 20 min at room temperature in the dark. Per measurement, 5000 cells were analyzed. Each experiment was performed in triplicates and repeated three independent times. The concentration of K048, K074, K075, and K203 oximes and TBHP (positive control) used in this assay corresponded to their IC_{50} values determined after 24 hours.

After 1 h of incubation (Figure S-1.) with tested oxime reactivators and TBHP as the positive control, the percentage of intact cells decreased significantly in all compounds in order K075, K203, K074, K048, and TBHP. The number of live cells was about 5.86, 8.43, 8.85, 9.71, and 11.6 % lower, respectively. A significant change in the percentage of cells undergoing early apoptosis was observed in oximes K048, K074, and K203. The amount of early apoptotic cells increased about 5.05, 2.45, and 1.51 %, respectively. On the other hand, the percentage of late apoptotic cells was significantly higher among all tested compounds (TBHP > K074 > K048 > K203 > K075) when compared to untreated control cells. The count of late apoptotic cells enlarged by 7.74, 4.71 , 4.17, 4.13, and 3.20 %, respectively. The statistical differences were also found in the percentage of necrotic cells in this time interval. The highest increment was observed in cells treated with TBHP (increase by 3.24 %), followed by a group of cells treated with oxime K074 (increase by 1.69 %). In the 4 h incubation interval, the percentage of live cells significantly decreased in all tested substances (TBHP > K048 > K203 > K074 > K075) by 14.6, 11.2, 8.8, 8.3, and 5.3 %, respectively. The most numerous populations of early apoptotic cells were observed in cells treated with K048 (increase by 5.72 %), followed by K074 (increase by 4.17 %) and TBHP (increase by 1.02 %). On the other hand, late apoptotic cells were significantly higher in TBHP, K048, K203, K074, and K075 (by 9.79, 4.35, 4.24, 3.47, and 2.69 %, respectively) compared to control cells. Also, a significant increase in necrotic cell population was found in all tested compounds in order TBHP, K203, K075, K048, and K074 (by 3.77, 1.74, 1.19, 1.17, and 0.67 %, respectively). After 24 h incubation (Figure 2.) with oxime AChE reactivators, the percentage of intact cells significantly decreased by 42.7, 45.6, 45.5, 46.2, and 46.6 % in TBHP, K048, K074, K075, and K203 groups when compared with control. Early apoptotic cells were significantly higher in TBHP, K048, K074, K075, and K203 groups (by 7.42, 18.2, 14.1, 12.4, and 13.3 %, respectively) than in control. The percentage of late apoptotic cells increased significantly by 31.6, 26.2, 31.3, 32.9, and 32.9 % in TBHP, K048, K074, K075, and K203 treated groups, respectively, when compared with control. Finally, the percentage of necrotic cells was significantly higher only in TBHP treated cells (increase by 3.66%).

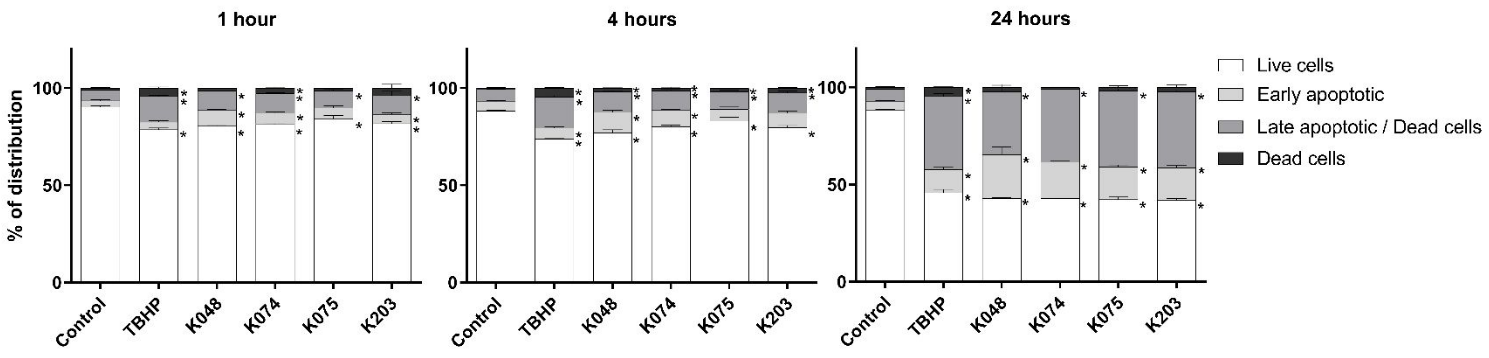

Fig. S-1 The percentage of intact (white column), early (light grey column), late apoptotic (dark grey column), and necrotic HepG2 cells (black column) determined using microcapillary flow cytometry after 1 h, 4 h and 24 h treatment with oxime acetylcholinesterase reactivators or TBHP (positive control). Control represents the cells incubated with an oxime-free culture medium. One-way analysis of variance (ANOVA) followed by Dunnet's multiple comparison test was used for statistical analysis by GraphPad Prism 9 software version 9.3.0. Data are expressed as means ± standard deviation (SD) of three independent measurements (n = 3). Significant differences between oxime-treated and untreated control groups ($p \leq 0.05$) and marked by asterisk (*).

3. Stability of oximes in DMEM medium at 37 °C over 24 hours

The stability of oximes K048, K074, K075, and K203 in DMEM medium, with concentrations corresponding to their IC50 values after 24 hours, was assessed after incubation at 37°C for 0, 0.5, 1, 2, 4, 6, 8, and 24 hours using HPLC-UV, following the chromatographic conditions described by Vanova et al. (2021)[13]. The results over the tested time intervals are presented in Figure S-2.

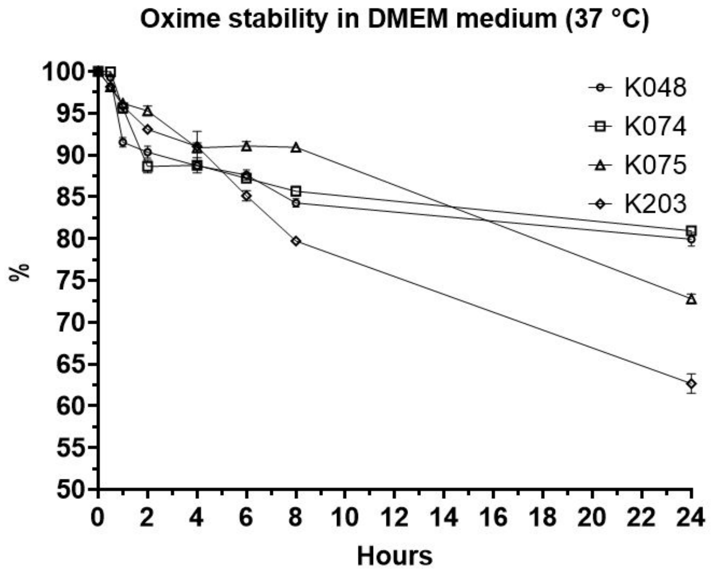

Fig. S-2 Data are expressed in percentage as means ± standard deviation (SD) of three independent measurements (n = 3).

4. Antioxidant activity of selected oxime reactivators of acetylcholinesterase

The concentration of oximes used for study of the antioxidant activity is shown in the Table S-2[39].

Table S-2. Oxime concentrations for antioxidant activity assay

compound	concentration [mmol/L]
K048	2.50
K074	1.25
K075	0.16
K203	0.16
